# The properties of TREM1 and its emerging role in pain-related diseases

**DOI:** 10.1186/s13041-025-01187-w

**Published:** 2025-02-26

**Authors:** Zhenzhen Fan, Longde Wang, Songtang Sun, Zhaoming Ge

**Affiliations:** 1 Department of Neurology, Lanzhou University Second Hospital, Lanzhou University, Lanzhou, 730000 China; 2 Department of Neurology, Henan Provincial People’s Hospital, Zhengzhou, 450003 China; 3 Expert Workstation of Academician Wang Longde, Lanzhou University Second Hospital, Lanzhou, 730000 China; 4 Gansu Provincial Neurology Clinical Medical Research Center, Lanzhou University Second Hospital, Lanzhou, 730000 China

**Keywords:** TREM1, Inflammation, Microglia, Pain

## Abstract

The TREM1 receptor, a member of the TREMs family, is expressed by myeloid cells and functions as an initiator or enhancer of the inflammatory response, playing a pivotal role in the regulation of inflammation. In recent years, it has been found that TREM1-mediated inflammatory response is involved in the regulation of pain-related diseases. This article provides an extensive review on the structural characteristics and distribution patterns, ligand, signaling pathways, inhibitors, and pathophysiological roles of TREM1 in pain disorders aiming to further elucidate its biological function and offer novel insights for clinical interventions targeting pain-related diseases.

## Introduction

Pain is an aversive sensation and affective experience that results from tissue and nerve damage or sensitization of the nervous system, representing one of the most prevalent motives for individuals to seek medical intervention [1]. The prevalence of chronic pain exceeds 30% globally, inflicting significant suffering on individuals and severely impacting their daily functioning [1, 2]. Elucidating the underlying mechanisms of pain is crucial for advancing the development of novel and efficacious therapeutic interventions, thereby playing a pivotal role in pain management and treatment [3, 4].

Under optimal physiological conditions, pain serves as a response to the activation of nociceptors and fulfills a protective role for the organism. However, when pain becomes intense and prolonged, it can be detrimental and often leads to significant suffering [5]. It is widely acknowledged among researchers that chronic pain is associated with alterations in neuronal plasticity within pain regulatory pathways, whereby such plasticity contributes to the peripheral sensitization of primary sensory neurons (trigeminal ganglion and dorsal root ganglion) as well as the central sensitization of pain-processing neurons (spinal cord and brain). Both peripheral and central sensitization serve as crucial pathophysiological mechanisms underlying chronic pain [6–8]. Numerous studies have confirmed that neuroinflammation mediated by microglia activation is involved in the changes of neuronal plasticity, and the crosstalk mechanism between microglia and neurons mediates the occurrence and maintenance of pain [9–11]. Therefore, characterizing microglial surface receptors and exploring the specific mechanisms of microglial activation will significantly enhance our comprehension of pain mechanisms.

Triggering receptors expressed on myeloid cells (TREMs) constitute a family of cell surface receptors that play a pivotal role in both innate and adaptive immunity [12]. TREM1, the inaugural member of the TREM family, was initially believed to be expressed on the surface of neutrophils and monocytes, serving as a mediator for inflammatory and immune responses [13]. TREM1 has a significant impact on promoting and amplifying immune-inflammatory responses by synergistically interacting with classical pattern recognition receptor signaling pathways, such as Toll-like receptors and NOD-like receptors. It is implicated in the pathogenesis of various infectious and non-infectious inflammatory diseases, autoimmune disorders, and even tumors, including sepsis, pneumonia, atherosclerosis, inflammatory bowel disease, rheumatoid arthritis, and hepatocellular carcinoma [14–17]. The current landscape includes ongoing clinical research on drugs targeting the TREM1 receptor, such as Nangibotide for septic shock [18] and PY159 for pancreatic cancer [18]. Recently conducted studies have demonstrated the expression of TREM1 in microglia within the central nervous system [19]. The activation of microglia, the exclusive glial cells in the brain, triggers the release of pro-inflammatory factors and neurotoxic mediators in response to various pathological conditions. The TREM1 facilitates the immune-inflammatory response of microglia, thereby contributing to the pathological mechanisms underlying various neurological disorders, such as ischemic stroke, subarachnoid hemorrhage, Parkinson’s disease, and Alzheimer’s disease [18, 20–22]. Our preliminary research has revealed that TREM1 expressed in microglia within the trigeminal nucleus caudalis (TNC) plays an essential part in mediating neuroinflammation, thereby facilitating central sensitization through activation of the NF-κB pathway and consequently contributing to the pathogenesis and progression of chronic migraine [23]. Considering the involvement of TREM1 in various inflammatory disorders and the crucial role of microglia-mediated inflammation in pain modulation, targeting TREM1 could emerge as a promising therapeutic approach for pain management. This article provides an overview of the current state of biological research on TREM1, including soluble TREM1 (sTREM1), with a particular focus on the role of TREM1 in pain and the remaining challenges.

## Biological characteristics of TREM1

### Structure and distribution of TREM1

The TREM1 gene is located on human chromosome 6p21 and mouse chromosome 17C3 [12]. However, the current research data regarding its crystal structure remain inconclusive with regards to the formation of homodimers by TREM1 [24]. TREM1 expressed on the human cell membrane is a type I transmembrane protein of 234 amino acids, consisting of a single extracellular immunoglobulin (Ig) -like domain of 184 amino acids, a transmembrane region with positively charged lysine residues, and a short cytoplasmic tail of 5 amino acids lacking any signaling motifs [25]. The recent studies have shown that the extracellular domain of TREM1 is capable of concentration-dependent homologous oligomerization, suggesting that aggregation of TREM1 at the membrane promotes its oligomerization. The transmembrane domain of TREM1 mediates the formation of a complex with the signal adaptor DNAX-activating protein 12 (DAP12), and in addition, DAP12 contributes to stabilizing TREM1 expression [26].

The initial belief was that TREM1 is primarily expressed on the surface of neutrophils and monocytes [13]. However, subsequent investigations have revealed its widespread expression in macrophages [27, 28], microglia [29], dendritic cells [30], osteoclasts [31], and platelets [32]. The expression of TREM1 has also been observed in non-myeloid cell types, including epithelial and endothelial cells [33]. Given its widespread expression across various cell types, TREM1 plays a pivotal role in the regulation of immunity and inflammation, rendering it a critical factor in cellular immune responses and inflammatory processes.

### TREM1 ligands

Currently, the specific ligand for TREM1 remains elusive, and there is a paucity of studies elucidating the signaling mechanism underlying receptor-ligand interaction. However, emerging evidence suggests that actin, high mobility group protein B1 (HMGB1), heat shock protein 70-kDa (HSP70), peptidoglycan recognition receptor 1 (PGLYRP1), and extracellular cold-induced RNA binding protein (eCIRP) are potential primary ligands for TREM1 [16, 34].

#### Actin

When the integrity of the cell membrane is compromised, intracellular actin is released into the extracellular milieu. As a pivotal damage-associated molecular pattern (DAMP), extracellular actin has been implicated in various clinical conditions including hepatic necrosis, septic shock, and adult respiratory distress syndrome [35]. TREM1 is expressed on platelets and regulates platelet function [32]. Recently, extracellular actin derived from platelets has been identified as a novel ligand for TREM1 in the context of sepsis [36]. Fu et al. [37] demonstrated that recombinant actin directly binds to the extracellular domain of TREM1, thereby enhancing an inflammatory response. This interaction can be inhibited by a selective inhibitor of TREM1, LP17, and was not observed in TREM1^-/-^ mice. Furthermore, they observed colocalization of actin with TREM1 in platelets and lung tissue slices from septic mice. Collectively, these findings suggest that actin serves as a ligand for TREM1 present on platelets.

#### HMGB1

HMGB1 was originally thought to be a non-histone DNA-binding molecule prevalent in mammals, mainly located in the nucleus, that stabilizes nucleosomes and allows DNA to bend to regulate target gene transcription [38]. In response to inflammation, HMGB1 is actively secreted by activated myeloid cells and released from necrotic or deceased cells [39]. HMGB1 can interact with a diverse range of immune sensors and receptors, including advanced glycation end product (RAGE) receptors and TLRs, to activate MAPKs and NF-κB pathways, thereby inducing inflammatory responses [40]. Wu et al. [41] demonstrated direct interaction between TREM1 and HMGB1 through immunoprecipitation and cross-linking analysis, establishing HMGB1 as the ligand for TREM1. There is evidence supporting a crosstalk mechanism between HMGB1 and pro-inflammatory cytokines that contributes to the development and maintenance of inflammatory diseases, potentially implicating them in the pathophysiological processes underlying chronic neuropathic pain resulting from neuroinflammation [42]. Wang et al. [43] established a neuropathic pain model of spared nerve injury (SNI) through nerve ligation and observed a significant upregulation in the expression levels of HMGB1, MIP-1α, CCR1, and CCR5 in the spinal cord of rats. Intrathecal administration of lidocaine effectively ameliorated hyperalgesia in SNI rats by suppressing HMGB1 expression and modulating the secretion of MIP-1α, CCR1, and CCR5. In a rat model of inflammatory pain induced by complete Freund’s adjuvant (CFA), Huang et al. [44] observed a significant up-regulation of HMGB1 and NF-κB expressions in the dorsal root ganglion (DRG). Triptolide was found to alleviate CFA-induced mechanical hyperalgesia by inhibiting the activity of the HMGB1/NF-κB signaling pathway.

In addition to studies on neuropathic pain and inflammatory pain, a substantial body of evidence indicates the involvement of HMGB1 in the regulation of cancer-related pain [45], visceral pain [46], fibromyalgia [47], and post-tissue injury pain [48]. It is noteworthy that scholars have engaged in a series of discussions regarding the role of HMGB1 in migraine. Dalkara et al. [49] initially employed the cortical spreading depression (CSD) model and observed an increase in neuronal release of HMGB1, which was subsequently found to be reduced upon inhibition of HMGB1 expression, leading to diminished CSD-induced trigeminal vascular activation, dural mast cell degranulation, and headache symptoms. Subsequently, Suzuki et al. [50] discovered that a single CSD stimulation did not induce a significant rise in HMGB1 levels or microglial activation; however, repeated CSD stimulations resulted in substantial release of HMGB1 and subsequent microglial activation, suggesting the potential involvement of HMGB1 in the chronic progression rather than acute attacks of migraine. With the advancement of research, scholars have successively confirmed the pivotal role of HMGB1 in familial hemiplegic migraine [51] and cluster headache [52]. A recent clinical study demonstrated a significant elevation in serum HMGB1 levels among migraine patients with drug overuse, potentially associated with trigeminal nerve sensitization [53]. The extensive attention from researchers towards the involvement of HMGB1 in pain, including migraine, has positioned targeting HMGB1 as a valuable therapeutic strategy for pain management. However, the role of TREM1/HMGB1 signaling in pain regulation remains unknown and necessitates further investigation.

#### HSP70

Hsp70, a ubiquitous molecular chaperone, exhibits potential anti-inflammatory properties and is extensively involved in diverse cellular processes related to protein folding and remodeling [54]. It plays an indispensable role throughout the entire protein life cycle, from synthesis to degradation, thereby maintaining protein homeostasis [55]. Studies conducted by Sharapova et al. [56] have demonstrated that HSP70 exhibits potential as a ligand for TREM1, and its interaction with TREM1 can trigger the activation of cytotoxic lymphocyte subsets. However, Wu et al. did not observe direct binding between HSP70 and TREM1 in their experimental study [41], which could potentially be attributed to the higher affinity of HMGB1 compared to HSP70. Nevertheless, investigations into the role of HSP70 in central nervous system disorders have revealed its ability to ameliorate neurodegeneration and safeguard neurons against various forms of stress-induced damage [19]. Considering the neuroprotective effect of HSP70 in central nervous system (CNS) diseases, as opposed to the pro-inflammatory effect of TREM1, it appears unlikely that HSP70 functions as a ligand for TREM1 in the CNS. Therefore, further investigations are warranted to establish its association with TREM1 in the CNS. In a study investigating migraines, the administration of nitroglycerin (NTG) significantly reduced the expression of HSP70 in the Trigeminal nucleus caudalis (TNC) in mice. However, exogenous administration of HSP70 notably alleviated mechanical hyperalgesia, photophobia, and anxiety-like behavior induced by NTG administration in mice [57]. These findings suggest that HSP70 may play a protective role in migraine pathogenesis. Notably, our previous research demonstrated proinflammatory effects of TREM1 in CM mice [23], thus reinforcing the notion that HSP70 is an unlikely ligand for TREM1 within the central nervous system.

#### PGLYRP1

PGLYRP1, a member of the peptidoglycan (PGN) recognition protein (PGRP) family, possesses antimicrobial properties [58]. Read et al. [59] demonstrated that PGLYRP1 could bind to TREM1 using surface plasmonic resonance (SPR) and flow cytometry techniques. A cross-sectional study of periodontitis revealed the role of TREM1 and PGLYRP1, with a clear overlap observed in factors affecting the expression of both genes, such as bleeding during exploration and the number of significant dental caries [60], yet the relationship between TREM1 and PGLYRP1 along with their associated signaling pathway remains unexplored. A recent study has found that the microglial innate immunity protein PGLYRP1 promotes neuroinflammation through the TREM1-SYK-ERK1/2-STAT3 axis, revealing the role of microglial PGLYRP1 as a mediator of neuroinflammation and a potential biomarker and therapeutic target for various neuroinflammatory diseases [61]. Given its expression in central nervous system microglia and its involvement as a neuroinflammatory mediator, PGLYRP1 holds promise as a therapeutic target for central nervous system pain.

#### eCIRP

Extracellular cold-induced RNA binding protein (eCIRP) is a damage-associated molecular pattern (DAMP) that facilitates inflammation and injury [62]. Wang et al. [63] employed SPR to elucidate a robust affinity between eCIRP and TREM1. Double immunofluorescence analysis demonstrated the effective co-localization of eCIRP and TREM1 on macrophages following stimulation with recombinant mouse CIRP. The interaction between eCIRP and TREM1 in macrophages was further validated using the FRET assay. This study represents the pioneering work unveiling eCIRP as an endogenous ligand for TREM1. Borjase et al. [64] demonstrated that inhibition of the interaction between TREM1 and eCIRP attenuates inflammation and enhances survival rates during liver ischemia/reperfusion. In investigations of neurological disorders, eCIRP induces neuroinflammation by activating the IL-6Ra/STAT3/Cdk5 pathway in neurons [65]. Further investigation is warranted to determine whether CIRP mediates inflammatory responses through activation of TREM1 in central nervous system diseases. Table 1 shows Ligands associated with TREM1.

**Table 1:** Ligands associated with TREM1

TREM1 ligands	Location	Immune functions	Reference
Actin	extracellular actin derived from platelets	enhance inflammatory response.	[36, 37]
HMGB1	actively secreted by activated myeloid cells; passively released by necrotic and dead cells	promote inflammatory reactions and tumorigenesis	[41]
HSP70	released by necrotic cells	induces activation of cytotoxic lymphocyte subsets	[56]
PGLYRP1	produced by neutrophils in antibacterial granules	promote neuroinflammation	[59, 61]
eCIRP	translocated from nucleus to cytoplasmic stress granules and subsequently released into the extracellular space under stressful conditions	promote neuroinflammation	[63, 64]

### TREM1 signaling pathway

**Fig. 1:**
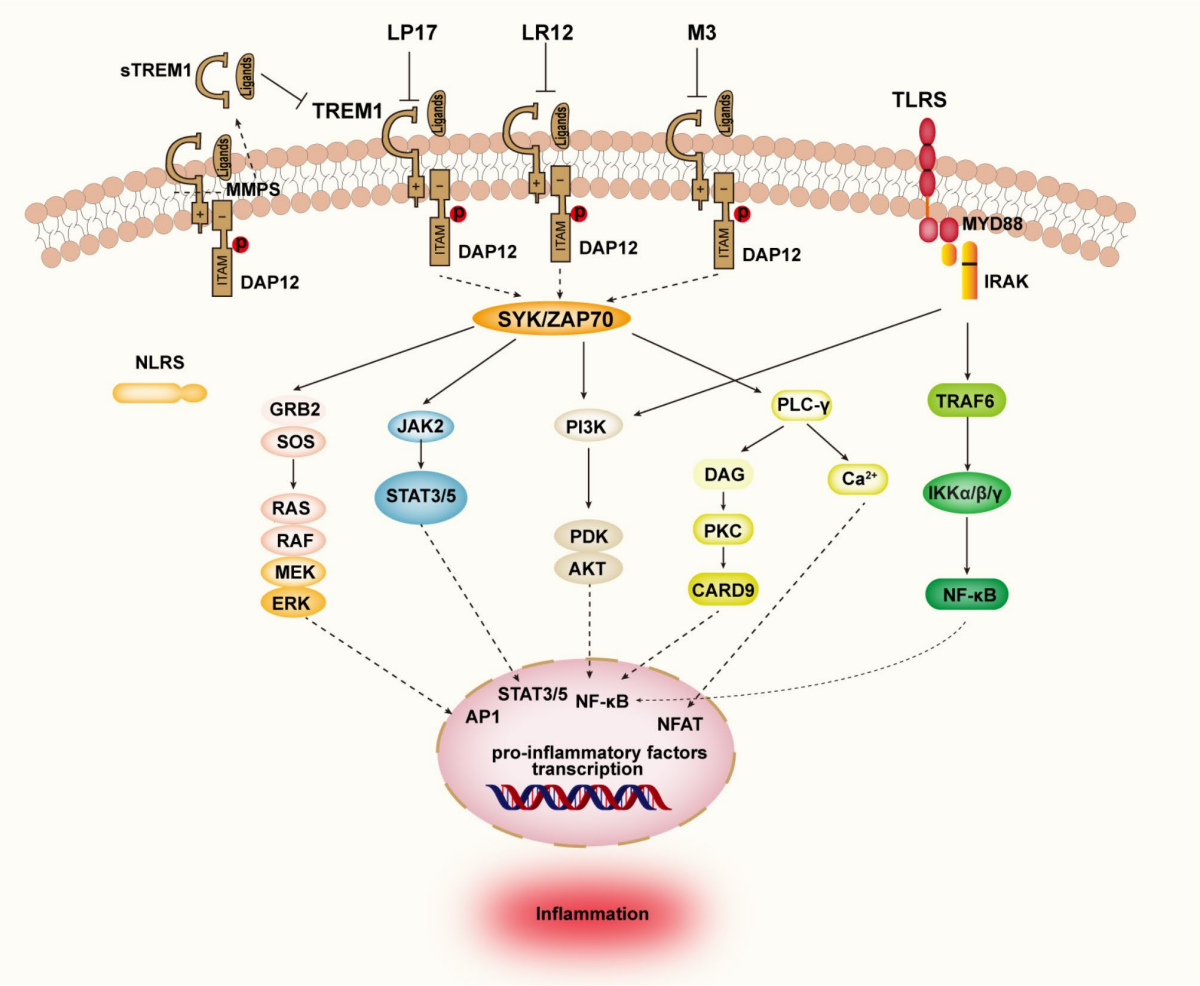
Schematic representation of the TREM1 signaling pathway. TREM1 and DAP12 form a stable TREM1-DAP12 complex through electrostatic interactions, which recruits SYK and ZAP70, leading to the phosphorylation of the immunoreceptor tyrosine activation motif (ITAM) of DAP12. This activation subsequently activates ERK, JAK/STAT, PI3K, and PLC pathways and regulates the transcription of inflammation-related genes. Additionally, PLC signaling regulates Ca^2+^ mobilization and gene transcription. TREM1 demonstrates synergy and interacts with TLRs

TREM1, a member of the immunoglobulin superfamily, lacks any signaling motif in its intracytoplasmic domain due to its specific structural characteristics; therefore, its signaling relies on binding with DAP12 [15]. The transmembrane lysine residue of TREM1, which carries a positive charge, interacts with the negatively charged aspartic acid residue of DAP12 to form the TREM1-DAP12 complex [66]. Upon receptor activation, phosphorylation of the immune receptor tyrosine activation motif (ITAM) in DAP12 occurs to recruit and activate spleen tyrosine kinase (SYK) and zeta-chain-associated protein kinase 70 (ZAP70) [67]. They trigger the activation of multiple downstream signaling pathways, including the PI3K/AKT pathway, Ras/ERK/MAPK pathway, NF-κB signaling pathway, and phosphorylation of phospholipase C. Consequently, this leads to elevated intracellular Ca^2+^ levels and the secretion of pro-inflammatory cytokines and chemokines [68, 69] (Fig. 1).

Studies have confirmed that activation of TREM1 can independently trigger the downstream inflammatory cascade [15]. Furthermore, studies have demonstrated synergy and interaction between TREM1 and TLRs. Activation of TLR4 upregulates the expression of TREM1, and co-activation of TREM1 and TLR4 leads to a significant increase in the release of proinflammatory cytokines and chemokines compared to TLR4 activation alone [70]. One proposed mechanism for this synergistic proinflammatory response is that TREM1 enhances the availability of downstream signaling molecules associated with TLR4, such as MyD88, CD14, NF-κB, and IκBα [71]. In LPS-stimulated mouse mononuclear macrophages (RAW), blockade of TREM1 does not affect the expression level of TLR4, but it reduces the expression of downstream signaling molecules and inflammatory cytokines [72]. A study demonstrated that upregulation of TREM1 led to increased expression of TLR4 in a mouse model of lung injury [73]. In addition, TREM1 and TLR2 can play a synergistic role at the cytokine induction level [74–76], but exactly how this synergistic effect is achieved remains to be further investigated. The findings suggest that the interaction between TREM1 and TLRs is intricate and intersecting in nature.

Nod-like receptors (NLRs), as a member of the pathogen recognition receptors (PRRs) family, possess the ability to detect both microbial infection and sterile tissue damage [77]. Furthermore, NLRs can synergistically collaborate with TLRs to regulate inflammatory and apoptotic responses [16]. TREM1 exhibits a synergistic effect on the inflammatory response triggered by NLRs recognition of peptidoglycan (PGN). Following moderate stimulation of TREM1 through NLRs ligands, activated TREM1 enhances NLRs signaling, induces NOD2 expression, and augments IL-1β and IL-6 production [78]. Further investigation is required to determine whether and how TREM1 interacts with NLRs in various diseases, such as pain, and to assess the significance of this interaction compared to other synergistic pathways involving TREM1. A comprehensive understanding of the signaling pathways associated with TREM1 could provide insights into its role in pain regulation and facilitate the development of novel therapeutic targets.

### sTREM1

The origin and role of sTREM1, a soluble form of TREM1, have sparked controversy. One hypothesis posits that the hydrolysis of TREM1 by metalloproteinases (MMPs) results in the shedding of its extracellular domain, thereby generating soluble TREM1 (sTREM1) [79, 80]. Alternatively, it has been proposed that sTREM1 is generated through alternative splicing of the TREM1 gene as a splice variant [81]. The endogenous decoy receptor sTREM1 competes with membrane TREM1 for binding to the same ligand, thereby inhibiting the signaling pathway of membrane TREM1 and subsequently reducing TREM1 activation and the release of pro-inflammatory cytokines [82].

Studies have shown that sTREM1 can be detected in plasma, cerebrospinal fluid (CSF), and bronchoalveolar lavage fluid in patients with inflammatory diseases [83–85]. High levels of plasma sTREM1 have been associated with sepsis, Alzheimer’s disease (AD), acute myocardial infarction, and subarachnoid hemorrhage [83, 86–88]. The investigation of sTREM1 as a potential biomarker for inflammatory diseases has garnered significant attention. Zhu et al.‘s study [82] demonstrated that the release of sTREM1 is contingent upon the activation and dimerization of TREM1, thus serving as an indicator of TREM1 activation. Importantly, our previous research initially confirmed the involvement of TREM1 in central sensitization among chronic migraine mice, highlighting that inhibiting its activity effectively mitigates hyperalgesia in these animals [23]. However, further extensive investigations are required to ascertain whether it can serve as a biomarker for pain or migraine patients. Due to its high degradability, the therapeutic utility of sTREM1 is limited; however, it can be employed for the development of specific inhibitors targeting TREM1 as scavenger receptors to hinder ligand binding to membrane-bound TREM1 and consequently attenuate TREM1 activation. The in vivo modulation of TREM1 by sTREM1 peptide represents a potentially valuable therapeutic strategy for treating inflammatory diseases, including pain.

### TREM1 inhibitors

#### LP17

LP17, a 17-amino acid peptide (LQVTDSGLYRCVIYHPP), shares the extracellular domain of mouse and human TREM1 and has been utilized as a potent inhibitor of TREM1 [89]. The mechanism of action of LP17 has been investigated, revealing its dual inhibitory effects on TREM1 activity. Firstly, it acts as a direct competitive inhibitor by binding to TREM1 and preventing its activation by the ligand. Secondly, it functions as a decoy receptor by binding to ligands of TREM1 prior to their ability to activate TREM1 [90]. Initially, scholars observed that LP17 can decrease the levels of TNF-α and IL-1β release in LPS-stimulated monocytes [91], providing preliminary evidence for its role. As research has progressed, subsequent studies in various animal models such as subarachnoid hemorrhage and cerebral infarction have further confirmed the biological function of LP17 in central nervous system diseases. Previous studies have demonstrated that LP17 effectively ameliorates brain damage induced by ischemic stroke through the reduction of neuroinflammation [92]. In a model of subarachnoid hemorrhage, LP17 mitigates the neuroinflammatory response by modulating microglial activation [93]. Recently, Li et al. [94] reported significant improvement in visceral hyperalgesia among patients with irritable bowel syndrome (IBS) following treatment with LP17, providing valuable evidence implicating TREM1 in pain regulation.

#### LR12

LR12, a small molecule peptide consisting of 12 amino acid sequences (LQEEDAGEYGCM) [95], was the pioneering TREM1 inhibitor to undergo clinical trials [96, 97]. Currently, there exists some controversy regarding the precise mechanism of action attributed to LR12. It appears that LR12 does not directly bind to TREM1 but functions as a decoy receptor by binding to the endogenous ligand of TREM1. Studies have demonstrated that in the absence of TREM1, LR12 fails to exhibit any biological effects [32]. Additionally, the activity of TREM1 may be inhibited by LR12 through its impact on oligomerization processes [26]. In a Phase 2a clinical trial, patients with septic shock received a continuous infusion of LR12 at a dosage of 3.0 mg/kg/hour within 24 h for up to 5 days without any reported adverse events [97], thus demonstrating its favorable safety profile. The efficacy of LR12 in mitigating cardiovascular dysfunction, organ failure, and inflammation induced by septic shock has been extensively investigated. In addition to sepsis, LR12 exhibits significant potential in various disease contexts. Recent studies have elucidated the role of LR12 in combating atherosclerosis [98]. Zhou et al. [99] demonstrated that LR12 effectively mitigates macrophage necrosis induced by acute lung injury (ALI) through inhibition of TREM1 activity, thereby ameliorating lung inflammation. Jiang et al. [100] confirmed that LR12 enhances the inflammatory response in thioacetamide (TAA)-induced acute liver failure (ALF) mice and promotes liver regeneration by stimulating macrophages to secrete CCL20 and activating the p38 MAPK pathway. Consequently, targeting LR12 may hold considerable therapeutic value for managing ALF. Studies investigating the effects of LR12 in sepsis, anti-atherosclerosis, lung disease, liver disorders, and other ailments have demonstrated its potent inhibition of TREM1 activity, leading to reduced inflammation and improved tissue or organ damage. However, limited research has been conducted on its role within the central nervous system, particularly in relation to the brain. Our findings indicate that LR12 effectively mitigates central sensitization in CM mice and alleviates hyperalgesia [23]. The favorable safety profile observed during clinical trials underscores LR12’s significant translational potential; nevertheless, further extensive investigations are warranted to ascertain its precise involvement in pain regulation.

#### M3

M3, a small peptide consisting of a 7-amino acid sequence (RGFFRGG), has been identified as a novel inhibitor of TREM1 through its binding to eCIRP, an endogenous ligand of TREM1 [63]. In an LPS-induced endotoxin mouse model, M3 significantly attenuates the expression levels of pro-inflammatory factors TNF-α and IL-6 [63]. Studies have demonstrated that M3 effectively mitigates sepsis-induced lung injury in mice by reducing serum levels of TNF-α and IL-6 [101]. Wang et al. [64] demonstrated that M3 exerts a suppressive effect on the expression of inflammatory markers (myeloperoxidase, macrophage inflammatory protein-2, cyclooxygenase-2) by disrupting the interaction between TREM1 and eCIRP, thereby enhancing the survival rate in liver ischemia-reperfusion injury. Siskind et al. [102] utilized recombinant CIRP to stimulate primary human glomerular endothelial cells (HRGEC), leading to a significant increase in the release of cytokines and sTREM1. Treatment with M3 notably down-regulated the expression levels of both cytokines and sTREM1, thereby inhibiting the activation of HRGEC cells. These findings indicate that M3 can effectively mitigate kidney injury by inhibiting TREM1.Denning et al. [103] demonstrated that M3 exhibits anti-inflammatory properties by inhibiting TREM1, thereby enhancing the survival rate in cases of intestinal ischemia-reperfusion injury. This study confirms the efficacy of M3 in blocking the interaction between eCIRP and TREM1, presenting a promising therapeutic strategy for mitigating intestinal ischemia-reperfusion injury. M3 is a new inhibitor, and a series of studies have shown its role in sepsis, liver ischemia-reperfusion injury, kidney injury, intestinal ischemia-reperfusion injury and other diseases, showing a bright therapeutic prospect. It can effectively reduce inflammation through inhibition of TREM1, and we speculate that it has potential application value in pain regulation. Further research is needed to confirm its role in pain disorders including migraine.

## The pivotal role of TREM1 in pain modulation

### TREM1 and neuropathic pain

Neuropathic pain (NPP) is a chronic secondary pain that results from damage to the peripheral or central somatosensory nervous system, leading to significant distress for patients. The majority of patients commonly experience intermittent or persistent sensations characterized by burning, tingling, and crushing, which may also give rise to induced pain [104]. Induced pain has the potential to propagate to other regions, with both peripheral and central sensitization playing a pivotal role in pain modulation [105].

Spinal cord injury (SCI) is a severe neurological disorder characterized by a high disability rate and limited recovery of neurological function, significantly impacting patients’ quality of life and imposing substantial social and economic burdens [106]. Li et al. [107] observed a significant up-regulation of TREM1 protein and mRNA expression levels in the spinal cord of a mouse model with SCI. However, the motor function impairment, mechanical and thermal pain hypersensitivity, as well as the elevation in inflammation-related factors induced by the SCI, were significantly reversed in TREM1 knockout mice. The inflammatory response and oxidative stress triggered by TREM1 is believed to contribute to the development of hyperalgesia subsequent to sensory nerve injury. Therefore, further investigation into the role of TREM1 in neuropathic pain models holds significant research value, and developing selective inhibitors that target TREM1 to inhibit inflammatory response signaling may be a promising therapeutic strategy for NPP treatment.

### TREM1 and visceral pain

Chronic visceral pain is a highly prevalent condition, affecting over 20% of the global population [108]. In addition to experiencing persistent pain, patients often endure emotional symptoms such as anxiety and depression [109]. Consequently, the pathophysiological mechanisms underlying chronic visceral pain are intricate, involving a cascade of processes spanning from microorganisms to the brain. The role of inflammation and immune response has gained increasing recognition, prompting scholars to shift their focus towards the central immune mechanism underlying chronic visceral hypersensitivity and depression-like symptoms subsequent to inflammation [110]. Zhang et al. [111] established a dextran sulfate sodium (DSS)-induced colitis model and observed higher upregulation of triggering receptor expressed on myeloid cells-1 (TREM-1) in the microglia of the anterior cingulate cortex (ACC) during the inflammatory phase in DSS mice. After inducing Trem1 gene knockout in mice (Trem1 KO) or administering a TREM-1 antagonist peptide (LP17), the expression of TREM-1 was downregulated, resulting in a decrease in visceral stimulation response and an elevation of pain threshold in mice. The aforementioned statement highlights the vital importance of TREM-1 in the inflammatory phase of colitis, indicating that inhibiting TREM-1 holds significant potential for alleviating visceral hyperalgesia in IBD. Li et al. [94] reported a significant increase in serum sTREM1 levels among patients with diarrhea-predominant irritable bowel syndrome (D-IBS), which were closely correlated with the severity and frequency of abdominal discomfort, including abdominal pain. Additionally, there was a notable association between TREM1 expression in macrophages within the colonic mucosa and the scores indicating severity and frequency of abdominal pain among D-IBS patients. These findings indicate that TREM1 plays an essential role in mediating the allergic response to visceral pain related to D-IBS, while elevated serum sTREM1 levels may be attributed to macrophage activation through the TREM1 signaling pathway. Consequently, targeting macrophage activation associated with TREM1 could potentially serve as a therapeutic strategy for managing visceral pain. In brief, the research mentioned above indicates that TREM1 contributes to the formation of visceral hypersensitivity by directly regulating macrophages in colitis mucosa or indirectly affecting astrocytes in the ACC.

### TREM1 and other pain

Low back pain is one of the leading causes of disability and causes a huge economic and social burden around the world, with disc degeneration (IVDD) being the main cause [112]. The pathological feature of IVDD is extracellular matrix (ECM) degradation caused by decreased nucleus pulposus (NP) cells and increased stroma-degrading enzymes [113]. Wang et al. [114] identified TREM1 as the gene exhibiting the most significant disparity between degraded and normal intervertebral discs. The expression of TREM1 protein is markedly upregulated in degenerated nucleus pulposus tissue, while in vitro experiments demonstrate that overexpression of TREM1 suppresses NP cell proliferation and enhances the expression of apoptosis and inflammatory factors. Liang et al. [115] employed single-cell sequencing to analyze distinct cell subsets of normal human disc nucleus pulposus (NP) cells and degraded NP cells, revealing the existence of a novel cell subset called chondrocyte 4, which showed significantly higher abundance in degraded NP cells. Subsequent functional characterization demonstrated that chondrocyte 4 exhibited elevated expression levels of TREM1 and other pain-related inflammatory genes. Therefore, these findings suggest that TREM1 may play a crucial role in the pathogenesis of disc degeneration, and targeting TREM1 holds promise for improving disc degeneration and reducing the incidence of low back pain.

The most prevalent causes of shoulder pain in adults are rotator cuff injury (RCI) and shoulder arthritis. Studies have demonstrated that persistent presence of inflammatory cells, pro-inflammatory cytokines, and angiogenesis in injured rotator cuff (RC) tendons triggers fibrotic changes leading to scarring [116]. Reducing inflammation constitutes a primary therapeutic strategy for patients with rotator cuff injuries and/or arthritis [116]. Agrawal et al. [117] employed flow cytometry to detect the expression of TREM1 in circulating immune cells and observed a significant upregulation of TREM1 expression in CD14^+^ monocytes compared to the non-arthritis group. Additionally, they found that arthritis patients exhibited significantly increased protein expression and mRNA levels of TREM1 in tendon cells compared to the non-arthritis group. Furthermore, there was a significant association between the expression of TREM1 in shoulder tendon tissue and the severity of glenohumeral arthritis. These findings suggest that TREM1 may play a role in modulating joint inflammatory response and contribute to the progression of shoulder pain.

### TREM1 and migraine

A Mendelian randomization study utilizing a pooled genome-wide association study (GWAS) of neurological diseases, employing genetic variants in plasma sTREM1 levels as instrumental variables, did not establish a causal relationship with migraine [85]. However, due to the limited sample size in this investigation, further research is warranted to elucidate the role of sTREM1 in migraine pathogenesis. By.

establishing a mouse model of CM, we have uncovered potential significance of TREM1 in the chronicity process through its mediation of microglial activation and inflammatory response for regulating central sensitization [23]. Our findings align with observed proinflammatory properties attributed to TREM1 in other central nervous system disorders. Given the scarcity of research on TREM1’s involvement in migraines, additional studies are imperative to determine its role in disease pathogenesis and chronicity. Table 2 shows TREM-1 expression in pain-related diseases.

**Table 2:** The expression of TREM-1 in pain-related diseases

Disease	Disease/Models	Species	Sample	Signaling Pathway	Expression	Reference
Neuropathic Pain	Spinal cord injury	mouse	spinal cord tissue	HO-1	TREM1 ↑	[107]
Visceral pain	the dextran sulfate sodium (DSS)-induced colitis model	mouse	serum anterior cingulate cortex (ACC)	ACCGlu neurons	s-TREM1 ↑ TREM1 ↑	[111]
	Irritable Bowel Syndrome with Diarrhea (D-IBS)	D-IBS patients	serum mucosal macrophages	-	s-TREM1 ↑ TREM1 ↑	[94]
Low back pain	disc degeneration pain	lumbar disc herniation	chondrocyte 4	-	TREM1 ↑	[115]
Shoulder pain	Rotator cuff injury	RCI patients	tendon tissue blood neutrophils	-	TREM1 ↑ TREM1 ↑	[117]
Migraine	chronic migraine	mouse	The trigeminal nucleus caudalis (TNC)	NF-κB	TREM1 ↑	[23]

### Summary and prospect

TREM1 has been extensively investigated in infectious and non-infectious diseases, as well as central nervous system disorders, due to its ability to enhance inflammatory/immune responses. Given the crucial role of neuroinflammation in pain modulation, the involvement of TREM1 in pain regulation has garnered increasing attention. However, limited studies have explored its specific role in pain modulation, and a comprehensive investigation into the origin and nature of TREM1 ligands and their contribution to pain regulation remains lacking. Elucidating the source and identity of these ligands, comprehending the molecular mechanisms underlying receptor-ligand interactions, and studying signal transduction regulation will facilitate our understanding of TREM1’s biological functions while also paving the way for novel targeted therapies based on endogenous ligands. Thoroughly characterizing TREM1 will advance our comprehension of its involvement in pain regulation and potentially provide therapeutic targets for clinical management.

## Data Availability

All data used in this article are available from the corresponding author on reasonable request if necessary.
