# Correction: The properties of TREM1 and its emerging role in pain-related diseases

**DOI:** 10.1186/s13041-025-01193-y

**Published:** 2025-03-20

**Authors:** Zhenzhen Fan, Longde Wang, Songtang Sun, Zhaoming Ge

**Affiliations:** 1 Department of Neurology, Lanzhou University Second Hospital, Lanzhou University, Lanzhou, 730000 China; 2 Department of Neurology, Henan Provincial People’s Hospital, Zhengzhou, 450003 China; 3 Expert Workstation of Academician Wang Longde, Lanzhou University Second Hospital, Lanzhou, 730000 China; 4 Gansu Provincial Neurology Clinical Medical Research Center, Lanzhou University Second Hospital, Lanzhou, 730000 China

**Correction: Molecular Brain (2025) 18:15** 10.1186/s13041-025-01187-w

Following publication of the original article [1], the authors identified an error in the order of the authors, corresponding authors and author contributions section.

The incorrect order of the authors: Zhenzhen Fan, Songtang Sun, Longde Wang and Zhaoming Ge

The correct order of the authors: Zhenzhen Fan, Longde Wang, Songtang Sun and Zhaoming Ge

The original corresponding author:

Zhaoming Ge

The correct list of corresponding authors:

Songtang Sun, Zhaoming Ge

The Author contributions section currently reads:

**Author contributions**

ZF designed and conducted the review, performed research summary, and drafted the manuscript. SS and LW provided valuable suggestions and contributed to the revision of the review. ZG supervised the writing progress and revised the manuscript. All authors read and approved the final version.

The Author contributions section should read:

**Author contributions**

ZF designed and conducted the review, performed research summary, and drafted the manuscript. LW and SS provided valuable suggestions and contributed to the revision of the review. ZG supervised the writing progress and revised the manuscript. All authors read and approved the final version.

The author group order, corresponding authors and author contributions section have been updated above and the original article [1] has been corrected.
